# Angiogenesis-Related Gene Expression Profile with Independent Prognostic Value in Advanced Ovarian Carcinoma

**DOI:** 10.1371/journal.pone.0004051

**Published:** 2008-12-29

**Authors:** Marta Mendiola, Jorge Barriuso, Andrés Redondo, Adrián Mariño-Enríquez, Rosario Madero, Enrique Espinosa, Juan Ángel Fresno Vara, Iker Sánchez-Navarro, Ginés Hernández-Cortes, Pilar Zamora, Elia Pérez-Fernández, María Miguel-Martín, Asunción Suárez, José Palacios, Manuel González-Barón, David Hardisson

**Affiliations:** 1 Department of Pathology, Hospital Universitario La Paz, Universidad Autónoma de Madrid, Madrid, Spain; 2 Translational Oncology Unit, Hospital Universitario La Paz, Universidad Autónoma de Madrid, Madrid, Spain; 3 Unit of Biostatistics, Hospital Universitario La Paz, Universidad Autónoma de Madrid, Madrid, Spain; 4 Fundación para la Investigación Biomédica del Hospital Universitario La Paz (FIBHULP), Madrid, Spain; 5 Department of Pathology, Hospital Universitario Vírgen del Rocío, Sevilla, Spain; Health Canada, Canada

## Abstract

**Background:**

Ovarian carcinoma is the most important cause of gynecological cancer-related mortality in Western societies. Despite the improved median overall survival in patients receiving chemotherapy regimens such as paclitaxel and carboplatin combination, relapse still occurs in most advanced diseased patients. Increased angiogenesis is associated with rapid recurrence and decreased survival in ovarian cancer. This study was planned to identify an angiogenesis-related gene expression profile with prognostic value in advanced ovarian carcinoma patients.

**Methodology/Principal Findings:**

RNAs were collected from formalin-fixed paraffin-embedded samples of 61 patients with III/IV FIGO stage ovarian cancer who underwent surgical cytoreduction and received a carboplatin plus paclitaxel regimen. Expression levels of 82 angiogenesis related genes were measured by quantitative real-time polymerase chain reaction using TaqMan low-density arrays. A 34-gene-profile which was able to predict the overall survival of ovarian carcinoma patients was identified. After a leave-one-out cross validation, the profile distinguished two groups of patients with different outcomes. Median overall survival and progression-free survival for the high risk group was 28.3 and 15.0 months, respectively, and was not reached by patients in the low risk group at the end of follow-up. Moreover, the profile maintained an independent prognostic value in the multivariate analysis. The hazard ratio for death was 2.3 (95% CI, 1.5 to 3.2; p<0.001).

**Conclusions/Significance:**

It is possible to generate a prognostic model for advanced ovarian carcinoma based on angiogenesis-related genes using formalin-fixed paraffin-embedded samples. The present results are consistent with the increasing weight of angiogenesis genes in the prognosis of ovarian carcinoma.

## Introduction

Ovarian carcinoma is the most important cause of gynecological cancer-related mortality in Western societies [1]. This is due to the fact that approximately 60% of cases are diagnosed at late stages of disease [2]. While patients with stage I disease have a 5 year overall survival of 90%, patients with stage III–IV have less than 20% [3], [4]. Despite the highly lethal nature of epithelial ovarian cancer, the clinical course of advanced disease is still difficult to predict in an individual patient.

Usually, the management of ovarian cancer involves surgery in order to achieve surgical cytoreduction followed by chemotherapy [5]. Combination platinum-paclitaxel chemotherapy has become a standard first line treatment for the advanced-stage disease [6]. Outcome is significantly improved with this regimen, thus 60 to 70% of patients initially respond to platinum-based chemotherapy, and approximately 40 to 50% achieve complete clinical remission. However even in this last group, at least half of the patients experience a recurrence within 4 years [7]. Classical parameters such as age at diagnosis, extent of disease (as expressed as FIGO stage), residual disease after surgery, and the histopathological features of the tumor are important prognostic factors [5]. Nonetheless, these prognostic factors are imperfect predictors of outcome. This could be due, in part, to the molecular genetic events underlying ovarian neoplasms'complexity and to the fact that they have yet to be understood. Therefore, a better understanding of the molecular pathways leading ovarian cancer is crucial for the establishment of new screening strategies and more effective therapies. Precise prognostic factors based on gene expression may identify patients who are more likely to die of disease despite the achievement reached in response to standard treatment. Previous studies attempt to develop accurate predictors of clinical outcome using genome-wide expression arrays. In this study, we have focused on one of the most important events involved in carcinogenesis: angiogenesis.

Angiogenesis is a complex and highly regulated process that consists of the development of new vessels originating from pre-existing ones. Described first by Judah Folkman in 1971, this process has been extensively documented as playing a main role in cancer commencement and progression. Solid tumors must acquire an angiogenic phenotype to grow beyond a critical size [8]. This process is regulated by the balance between positive and negative inputs in the local environment. Regarding ovarian cancer, increased angiogenesis is associated with rapid recurrence and decreased survival. Moreover, targeting of *Vascular Endothelial Growth Factor* (*VEGF*) has produced good results in early clinical trials, thus suggesting the critical role of this process in the development and maintenance of ovarian carcinomas [9], [10]. Although there are many studies on the implication of different angiogenic factors in ovarian carcinoma, these are not based on angiogenic profiles related to survival or to treatment response. In this study, we have evaluated an 82 angiogenesis-related gene set in a series of 61 advanced ovarian carcinoma samples by quantitative real-time PCR (qRT-PCR). This technique, considered the gold standard for measurement of gene expression, has the ability to analyze very small mRNA fragments makes this assay feasible for studies with formalin-fixed paraffin-embedded samples, in which the RNA is moderately or even highly degraded.

The main goal of this study was, therefore, to identify an angiogenesis-related gene expression profile with prognostic value in advanced ovarian carcinoma (AOC) patients.

## Methods

### Patient data

We included 61 non-consecutive FIGO stage III/IV ovarian carcinomas from patients treated at the Hospital Universitario La Paz (Madrid, Spain) between February 1996 and December 2003. All patients underwent a baseline CT scan and exploratory laparotomy for diagnosis, staging, and debulking when feasible. All patients received a platinum/taxane-based chemotherapy for at least 6 cycles in total. Surveillance of patients in the study included periodical physical examination and CT scan performed three-monthly during and after the completion of the treatment. Follow-up data were obtained by retrospective chart review. Approval for the study was obtained from the Local Ethics Committee (Comité Ético de Investigación Clínica del Hospital Universitario La Paz, ref. PI-423). According to our Local Ethics Committee it was not necessary to obtain a verbal or written consent for publication from the patients included in this study considering that this is a retrospective study based on formalin-fixed, paraffin-embedded tumor samples obtained between February 1996 and December 2003.

### Clinical definitions

Patients were staged according to the International Federation for Gynecology and Obstetrics (FIGO) classification. Optimal debulking was defined as ≤1 cm (diameter) residual disease. A complete response (CR) was defined as absence of all clinical/radiographic evidence of disease. In addition, a second-look laparotomy (SLL) was performed on most of the patients having achieved a CR after planned treatment, and all of them who were optimally debulked. In patients that after the treatment planned achieved a CR and did not accept a SLL, or whether this procedure was not feasible, and also in patients with a partial response, a second CT scan was performed one month after the first evaluation to confirm the response. Progression-free survival (PFS) was defined as the time interval between the start of the treatment and the first confirmed sign of disease recurrence or progression. Overall survival (OS) was defined as the time interval between the start of the treatment and the date of death or end of follow-up.

### RNA extraction and reverse transcription

To determine the gene expression patterns in tumor biopsies, tissue sections previously stained with hematoxylin and eosin were reviewed by an experienced pathologist. Eligible samples included at least 80% of tumor cells and no large necrotic areas. Four to eight 4-µm sections were used for RNA isolation, with the Masterpure RNA Purification Kit (EPICENTRE Biotechnologies, Madison, WI, USA) according to the manufacturer's instructions. One µg of total RNA was used for cDNA synthesis according to the protocol provided with the High Capacity cDNA Reverse Transcription kit (Applied Biosystems, Foster City, CA, USA).

### Gene selection and qRT-PCR

The selection of genes included in the study was as follows: first, we selected genes previously described in ovarian carcinoma samples or cell lines with a role in the carcinogenesis process. Second, we also included genes described as modulators of angiogenic process, but not previously described in ovarian carcinoma. Thus, eighty two genes were selected by literature data mining. Specific TaqMan Gene Expression assays for each gene were selected and gene expression was determined by qRT-PCR with TaqMan Low Density Arrays (TLDA) in an ABI PRISM 7900 HT Sequence Detection System (Applied Biosystems). Each TLDA was configured with 96 genes by duplicate for two samples. Fourteen housekeeping genes selected by GeNorm Analysis software, as previously described [11], were added to normalize the raw data.

Ct values, defined as the point at which the fluorescence rises above the background fluorescence [12], were calculated with SDS 2.2 software (Applied Biosystems). We applied a normalization factor based on the geometric mean of the best-performing housekeeping genes.

### Statistical Analysis

Quantitative data were described by median (minimun-maximun) and qualitative data as absolute frequencies and percentages. Associations between categorical variables were evaluated with the χ^2^ test and the Fischer's exact test when appropriate. All deaths observed in the data set were cancer related, meaning that OS is equivalent to cancer-specific survival for purposes of this analysis. OS and PFS curves were generated by the Kaplan-Meier method, and differences between survival curves were assessed for statistical significance with the log-rank test.

The Cox regression analysis was used to build multiple models based on the combination of significant genes. The Akaike Information Criterion based selection was used to find the most accurate one [13]. Protective genes were defined as those associated with a hazard ratio (HR) of less than 1; risk genes were defined as those associated with a hazard ratio of more than 1. Two risk groups (low and high risk) were then assigned according to the expression level of the genes included in the profile.

Considering that all samples were used to generate the model, the estimated accuracy of the gene profile could be over-optimized. To correct this bias, we performed a leave-one-out cross validation (LOOCV) [14]. The model accuracy was determined by using the receiver operating characteristics (ROC) curves to calculate its sensitivity and specificity. The Kaplan-Meier method coupled to a log-rank test was used to generate survival curves. Multivariate analysis for confounding factors was carried out by using Cox proportional hazards regression with categorical or continuous variables as appropriate. For this analysis, gene profile was considered a binary category (low risk or high risk). P values of all statistics tests were two sided.

SAS 9.1, Enterprise Guide 3.0 and SPSS (version 9.0; SPSS Inc Chicago, IL, USA) packages were used for statistical tests. LOOCV was performed using R language version 2.2 with the Design Software package version 2.0.

## Results

The median age at diagnosis was 53 years (range, 21 to 82 years). All patients had advanced disease (FIGO stages III/IV). Most of them had FIGO stage III (51, 83.6%), grade 3 tumors (35, 57.4%), and serous histology (42, 68.9%). Thirty one percent of patients were optimally cytoreduced after initial surgery (≤1 cm residual diameter disease). All received postoperative taxane-platinum based combination therapy and 52 patients (85.2%) achieved an initial response to this therapy. The median follow-up was 44 months (range, 1 to 127 months), with a median OS of 40 months (95% CI 25.4–54.8) and a median PFS of 17 months (95% CI 13.7–20.6) for the entire group.

Optimal surgery and response to initial treatment were predictors of survival in the univariate analysis. None of the other clinico-pathological characteristics (FIGO stage, age and histopathology) showed statistically significance in the univariate analysis (Table 1).

**Table 1:** Univariate and multivariate analysis for PFS and OS.

	Univariate p & HR (95% CI) values				Multivariate p & HR (95% CI) values			
	PFS		OS		PFS		OS	
**Debulking status (<1 cm vs ≥1 cm)**	0.023	0.45 (0.23–0.91)	0.003	0.27 (0.12–0.63)	0.547	0.79 (0.36–1.71)	0.625	0.75 (0.23–2.37)
**Age (≤40 vs >40)**	0.395	N/A	0.556	N/A	N/A	N/A	N/A	N/A
**Stage (III vs IV)**	0.985	N/A	0.867	N/A	N/A	N/A	N/A	N/A
**Histology (serous vs other)**	0.339	N/A	0.807	N/A	N/A	N/A	N/A	N/A
**Grade (1 vs 2–3)**	0.148	N/A	0.338	N/A	N/A	N/A	N/A	N/A
**Profile (low vs high risk)**	<0.001	1.03 (1.02–1.04)	<0.001	2.72 (1.85–4.00)	<0.001	1.03 (1.02–1.04)	<0.001	2.25 (1.58–3.20)
**Response to treatment (complete plus partial vs absence)**	<0.001	0.24 (0.11–0.51)	<0.001	0.08 (0.03–0.19)	0.497	0.76 (0.35–1.67)	0.041	0.054 (0.03–0.889)

P, hazard ratio (HR) and 95% confidence interval (95% CI) values from unadjusted and adjusted Cox Regression. Hazard ratios for non significant values are noted as N/A.

### Gene expression analysis and the development of the prognostic profile

In order to evaluate reference gene stability among samples, C_t_ values of 14 housekeeping genes were imported into GeNorm v.3.4 [11]. This software was developed to identify which ones and how many control genes should be used from a larger list of putative ones, based on the lowest variation among samples. With this approach, we obtained a combination of five genes (*18S*, *ACTB*, *B2M*, *GAPDH* and *GUSB*) which was the most appropriated to normalize the results. The remaining 9 genes (*HMBS*, *HPRT1*, *IPO8*, *PGK1*, *PPIA*, *RPLP0*, *TBP*, *TFRC* and *UBC*) were excluded from the study because of the high variability between samples. We calculated a normalization factor based on the geometric mean of those genes, and we applied it to expression values of all the other genes tested.

Hazard ratios from univariate Cox regression analysis were used to determine which genes were associated with OS. The approach to model building for prediction in survival analysis was based on the combination of stepwise regression, Akaike information criteria (AIC), and the best subset selection: Stepwise-AIC-Best Subsets approach. As all the samples were used to generate the model, LOOCV was applied to avoid bias related to the accuracy overestimation of the model.

**Figure 1:**
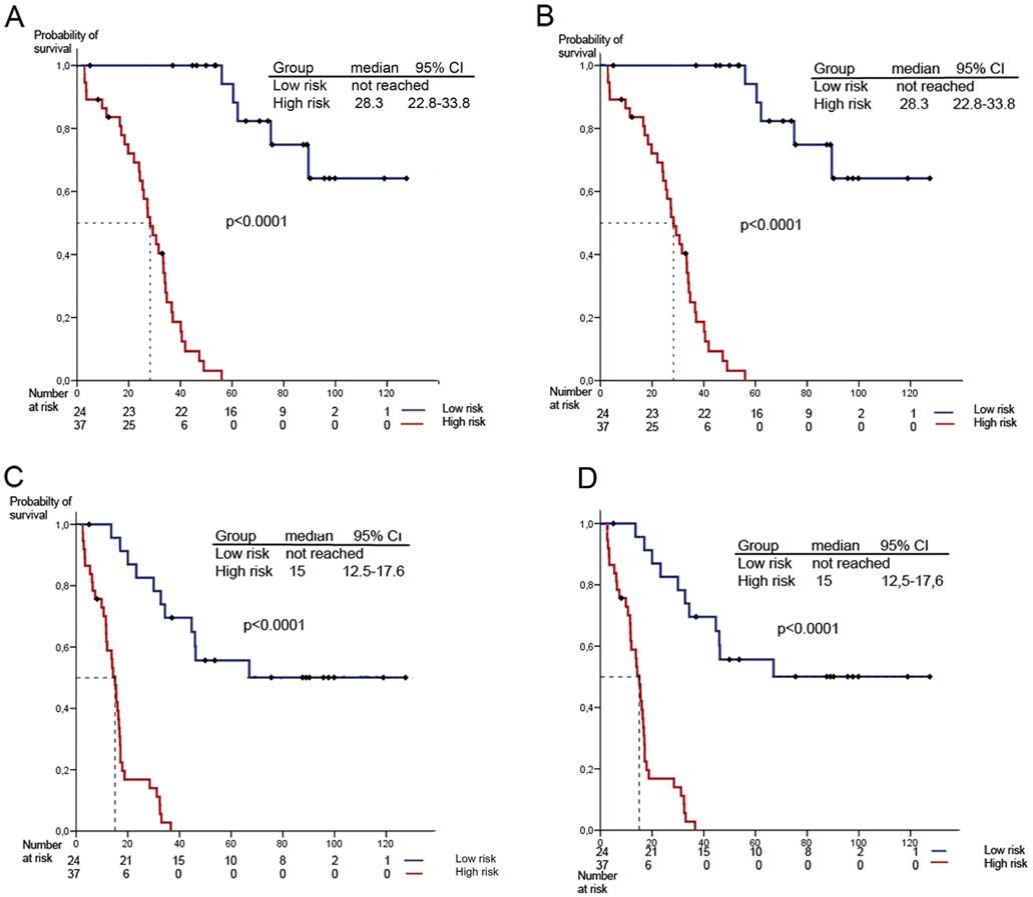
Kaplan-Meier OS curve of low risk *versus* high risk group (A). Kaplan-Meier curve after LOOCV (B). Kaplan-Meier PFS curve of low risk against high risk (C). Kaplan-Meier curve after LOOCV (D).

We established a 34 gene profile related to OS of ovarian carcinoma patients (Table S1). Hazard ratios from the univariate Cox regression analysis showed that the levels of expression of 34 genes correlated with OS: 17 were protective genes (hazard ratio less than 1) and 17 were risk genes (hazard ratio of more than 1). With this profile, we were able to distinguish between two groups of patients (low risk and high risk) with a sensitivity of 87.2% and a specificity of 86.4% after LOOCV. According to this classification, the high-risk group had a median OS of 28.3 months (95% CI, 22.8 to 33.8) (p<0.0001) whereas median OS was not reached in the low risk group at the end of follow-up (HR: 2.72, 95% CI: 1.85–4.00, p<0.0001) (Figure 1A and B).

The prognostic profile was also used to asses PFS (Figures 1C and D). Median PFS for the high risk group was 15.0 months (95% CI, 12.5 to 17.6) (p<0.0001) and was not reached in the low risk group (HR: 1.03, 95% CI, 1.02–1.04, p<0.001).

**Figure 2:**
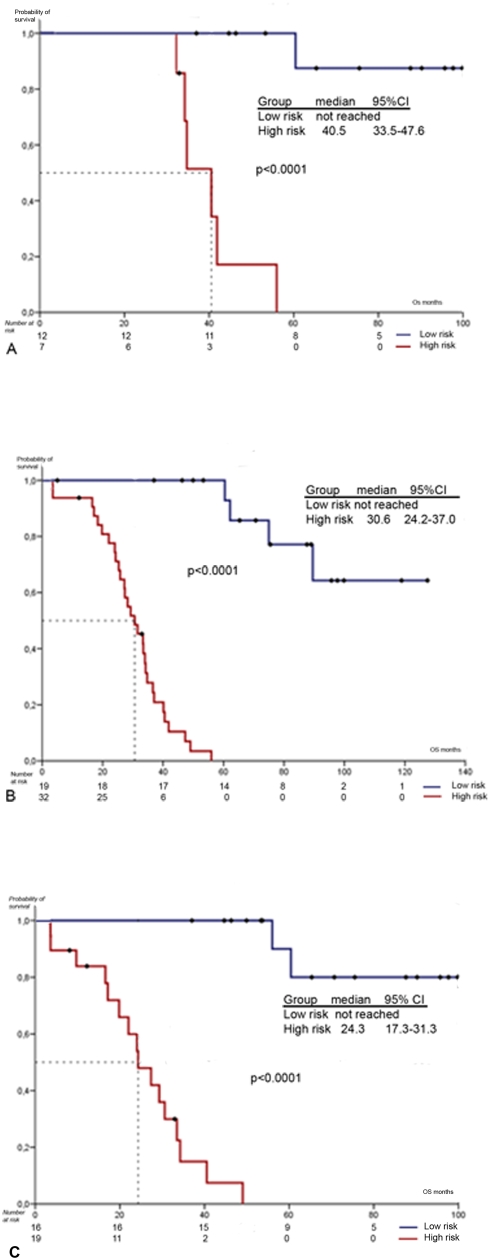
Kaplan-Meier OS curve (low risk versus high risk) in optimally debulked subgroup (A), in FIGO stage III subgroup (B), and in differentiation grade 3 subgroup (C).

Figure 2 shows the Kaplan-Meier analysis as a function of gene profile for homogeneous subsets of patients. Our profile maintained its significance even in the group of responders. Thus, the analysis amongst patients who achieved a partial or complete response showed a median OS for the high risk group of 33.3 months (95% CI, 27.8 to 38.9) whereas it was not reached for the low risk group (p<0.0001). Therefore, the profile provided excellent discrimination of survival curves for these patients' subsets.

The univariate analysis showed a statistically significant association between the angiogenesis-related gene expression profile and the debulking status and the response to treatment (complete or partial response vs absence of response) (Table 2).

**Table 2:** Relationship between the angiogenesis-related gene profile and clinico-pathological parameters.

	Low risk group		High risk group		p
	Nr	%	Nr	%	
**Age**					
**≤40**	2	3.3	5	8.2	0.694
**>40**	22	36.0	32	52.5	
**Grade**					
**1**	3	5.0	2	3.3	0.380
**2–3**	21	35.0	34	56.7	
**Stage**					
**III**	19	31.2	32	52.4	0.495
**IV**	5	8.2	5	8.2	
**Histology**					
**Serous**	17	27.9	25	41.0	0.788
**Other histology**	7	11.5	12	19.6	
**Debulking status**					
**Optimal**	12	25.5	7	14.9	0.019
**Suboptimal**	8	17.0	20	42.6	
**Response**					
**Yes**	24	39.3	28	45.9	0.007
**No**	0	0	9	14.8	

P values: χ^2^ for histology and debulking status, Fischer's exact test for stage, grade and age.

### Multivariate analysis

The prognostic profile maintained independent prognostic significance in multivariate analysis when correcting for debulking status and the response to therapy. The HR for death in low risk group versus the high risk group was 2.25 (95% CI, 1.58 to 3.20; p<0.001) (Table 1). Debulking status was not independently associated with OS, and the achievement of a response had a HR of 0.054 (95% CI, 0.03 to 0.889, p = 0.041). The results were similar when related to the PFS, with a HR for the prognostic profile of 1.03 (95% CI, 1.02 to 1.04; p<0.001). In this setting, neither the debulking status nor the responses were independent prognostic factors.

## Discussion

Microarray technologies have already provided valuable expression data in the classification of ovarian cancers based on gene profiling [15]–[19]. However, the selection of patients for new therapeutic strategies remains a challenge [20], [21].

Low density arrays combine the capacity to measure the expression of many genes in a single sample (such as microarrays approach), while retaining the sensitivity and quantitative range offered by qRT-PCR.

An important limitation of high throughput techniques is the high quality requirements of starting RNA. The improvement of isolating RNA kits and the intrinsic characteristics of the qRT-PCR approach has overcome the problem of using formalin-fixed, paraffin-embedded samples. The ability of real-time RT-PCR to test the expression of very small mRNA fragments makes this assay affordable for studies with these kind of samples, in which the RNA is moderately or even highly degraded, and yet it still produces reliable results [22], [23]. Moreover, RT-PCR may be more feasible in the clinical setting than microarray-based technologies due to the need for specialized laboratory facilities and complex statistical analysis [15]–[19].

We tried to obtain the maximum biological plausibility analyzing the expression of a group of genes involved in the same biological process by studying pathways implicated in the angiogenic process. To determine the gene expression patterns, RNA was extracted from 61 samples. We used Cox regression analysis based on the combination of significant genes for model selection. The Akaike Information Criterion was used to find the most accurate one [13]. Rather than splitting data into test and validation sets, we performed a cross-validation, that uses repeated data-splitting to prevent model overfitting and to generate accurate estimates of model coefficients, being a compelling statistical technique for model validation [14].

In the present study, the angiogenesis-related gene profile provided independent prognostic information for OS outcome in patients with advanced epithelial ovarian cancer. In addition, the profile allowed the differentiation of two groups with different PFS outcome (Figure 1).

The prognostic power of the angiogenesis profile was independent on its association with the debulking status, which is the best established prognostic factor in advanced ovarian carcinoma. Moreover the profile was the only variable that achieved prognostic significance in both the OS and PFS multivariate analysis in our study (Table 1). Even though there was an association between response to treatment and the profile (p = 0.007), the profile maintained a strong prognostic significance when applied to the homogeneous group of patients with chemosensitive disease, thus confirming its independent value. This prognostic profile may have important clinical implications, as a tool to complement clinical risk-stratification of ovarian carcinoma patients.

Many of the genes included in the prognostic profile are known to confer a poor outcome for patients with advanced epithelial ovarian cancer (Table S1). One of these genes is the *VEGF*. Several *in vitro* studies showed that *VEGF* is crucially involved in various steps of ovarian carcinogenesis [24], [25]. More recently, inmunohistochemically detected VEGF overexpression and elevated serum levels of *VEGF* were shown to be associated with an impaired prognosis [26]–[29]. As expected, wild-type *BRCA2* was a risk gene. It is well known that *BRCA* deficient cells are particularly sensitive to platinum compounds [30], [31]. *PDGFRα* has been linked to poor prognosis and aggressive tumor characteristics [32], [33]. Furthermore, a previous report showed that the presence of this receptor at the mRNA level suggested that an autocrine or paracrine loop might be functional in epithelial ovarian cancer [34]. Moreover, *PDGFRα* was a component of the molecular profile reported by Spentzos et al [15]. Recently, *ID1* up-regulation was correlated with poor prognosis [35]. Thus, the presence of this gene in our profile underlined the importance of this family of proteins involved in angiogenesis.

Our profile also included some genes that are well known for their involvement in angiogenesis but, otherwise, their implication in prognosis of AOC has not been yet well established. Amongst them, *EPHB2* has only been reported twice as a biomarker with negative prognostic value in ovarian carcinoma [15], [36]. One of the matrix metalloproteinases inhibitor family involved in the epithelial to the mesenchymal transition, *TIMP1*, emerged in our profile as a protective factor. The presence of *EPOR* as a risk factor is another pathway to be explored in further studies. A recent study has described a paracrine mechanism of growth related to EPOR/EPO interaction, apparently independent of exogenous EPO [37]. These interesting observations about the role of these genes in ovarian cancer remain to be undoubtedly established and cannot be conclusively derived from this descriptive study.

An important advantage of our profile is that it has been developed from formalin-fixed, paraffin embedded tissue thus allowing its use in a widespread clinical setting without the need of frozen tumor tissue, which is usually only available in tertiary referral hospitals or research centers that have frozen tissue banks. Although our series is quite homogeneous, the fact that our study analyzed a relatively small number of samples may seem a limitation. Notwithstanding the majority of the profiling studies previously published on ovarian cancer used a similar number of cases [16], [38].

Our profile could be used as prognostic tool to enable clinicians to identify those high risk patients who will potentially benefit from alternative drug combinations. Moreover, due to the successful introduction of the antiangiogenic drugs in resistant advanced epithelial ovarian cancer, we could consider patients with a high risk to benefit from a combination of platinum-taxane chemotherapy with a novel antiangiogenic drug. Nevertheless, although our data suggest the potential utility of this approach, the prognostic value of our qRT-PCR based angiogenesis-related gene expression profile should be further evaluated in prospective studies of patients with advanced epithelial ovarian cancer.

## Supporting Information

Table S1 Profile genes associated with overall survival.
(0.06 MB DOC)
