# Supplementary material for: Angiogenesis-Related Gene Expression Profile with Independent Prognostic Value in Advanced Ovarian Carcinoma
Source: PLoS One. 2008 Dec 29;3(12):e4051. doi: 10.1371/journal.pone.0004051 (PMC2605264; doi:10.1371/journal.pone.0004051)
Supplement: Table S1 — Profile genes associated with overall survival. (0.06 MB DOC) [file pone.0004051.s001.doc]

**Supplementary Table 1. Profile genes associated with overall survival.**

| **Official Symbol** | **Gene Name** | **Expression in the High Risk group** | **p value** |
| --- | --- | --- | --- |
| ***AURKC*** | aurora kinase C | Decreased | 0.002 |
| ***BRCA2*** | breast cancer 2, early onset | Increased | 0.044 |
| ***CD34*** | CD34 molecule | Increased | <0.001 |
| ***CXCL1*** | chemokine (C-X-C motif) ligand 1(melanoma growth stimulating activity, alpha) | Increased | 0.003 |
| ***CXCL12*** | chemokine (C-X-C motif) ligand 12 (stromal cell-derived factor 1) | Decreased | 0.003 |
| ***EDN1*** | endothelin 1 | Decreased | 0.002 |
| ***EDNRA*** | endothelin receptor type A | Decreased | 0.001 |
| ***EDNRB*** | endothelin receptor type B | Increased | 0.002 |
| ***EFNB2*** | ephrin-B2 | Decreased | 0.003 |
| ***EGF*** | epidermal growth factor | Increased | 0.003 |
| ***EPHB2*** | EPH receptor B2 | Increased | 0.009 |
| ***EPHB4*** | EPH receptor B4 | Decreased | 0.002 |
| ***EPOR*** | erythropoietin receptor | Increased | <0.001 |
| ***ETS1*** | v-ets erythroblastosis virus E26 oncogene homolog 1 (avian) | Increased | <0.001 |
| ***FGF2*** | fibroblast growth factor 2 (basic) | Decreased | 0.003 |
| ***FLT1*** | fms-related tyrosine kinase 1 (vascular endothelial growth factor/vascular permeability factor receptor) | Increased | 0.002 |
| ***ID1*** | inhibitor of DNA binding 1, dominant negative helix-loop-helix protein | Increased | 0.020 |
| ***IL6*** | interleukin 6 (interferon, beta 2) | Decreased | <0.001 |
| ***IL8*** | interleukin 8 | Decreased | 0.006 |
| ***KLK6*** | kallikrein-related peptidase 6 | Decreased | <0.001 |
| ***MMP3*** | matrix metallopeptidase 3 (stromelysin 1, progelatinase) | Decreased | 0.002 |
| ***MMP7*** | matrix metallopeptidase 7 (matrilysin, uterine) | Increased | 0.001 |
| ***NOS3*** | nitric oxide synthase 3 (endothelial cell) | Decreased | 0.002 |
| ***NRP1*** | neuropilin 1 | Decreased | 0.039 |
| ***PDGFRB*** | platelet-derived growth factor receptor, beta polypeptide | Decreased | <0.001 |
| ***PDGFRA*** | platelet-derived growth factor receptor, alpha polypeptide | Increased | 0.002 |
| ***PLAU*** | plasminogen activator, urokinase | Decreased | 0.003 |
| ***PLAUR*** | plasminogen activator, urokinase receptor | Increased | <0.001 |
| ***ROBO1*** | roundabout, axon guidance receptor, homolog 1 (Drosophila) | Increased | <0.001 |
| ***TGFB1*** | transforming growth factor, beta 1 | Decreased | <0.001 |
| ***TIMP1*** | TIMP metallopeptidase inhibitor 1 | Decreased | <0.001 |
| ***TP53*** | tumor protein p53 | Increased | 0.001 |
| ***VEGF*** | vascular endothelial growth factor A | Increased | 0.004 |
| ***VEGFB*** | vascular endothelial growth factor B | Increased | 0.001 |
